# Multi-Scale Modeling and Damage Mechanisms of Asphalt Pavements Under Coupled Salt–Thermal–Mechanical Effects

**DOI:** 10.3390/ma18102337

**Published:** 2025-05-17

**Authors:** Jin Ma, Jiaqi Chen, Mingfeng Tang, Yu Liu

**Affiliations:** 1 Guangxi Communication Investment Technology Co., Ltd., Nanning 530001, China; mailmajin@163.com (J.M.); mailmingfeng@163.com (M.T.); hitrain@163.com (Y.L.); 2 Guangxi Expressway Maintenance Engineering Technology Research Center, Nanning 530001, China; 3 Key Laboratory of Intelligent Road Maintenance in Guangxi Transportation Industry, Nanning 530001, China; 4 Department of Civil Engineering, Central South University, Changsha 410075, China

**Keywords:** salt erosion, asphalt pavement, cohesive zone model, thermal properties, finite element analysis

## Abstract

Salts can have detrimental effects on asphalt pavements, leading to permanent damage that compromises their durability and sustainability. This study investigates the damage mechanisms of asphalt pavements under coupled salt–thermal–mechanical effects using multi-scale modeling. Pull-off and semicircular bending (SCB) tests were conducted to determine material parameters and validate numerical models. Experimental data demonstrated that after 48 h of salt treatment at −10 °C, specimens exhibited reductions in cohesive strength ranging from 23.5% to 26% and adhesive strength decreasing by 25% to 44% compared to untreated controls. More severe degradation was observed at 0 °C, with cohesive strength diminishing by up to 63.8% and adhesive strength declining by up to 71.6%. A multi-scale finite element (FE) pavement model incorporating cohesive zone modeling (CZM) was developed to simulate damage behavior within asphalt concrete. Salt diffusion analysis revealed limited penetration depth within short exposure periods, and results showed that salt penetration reached only about 10 mm into the pavement layers after 48 h. Results indicated significant reductions in adhesive and cohesive strengths due to salt exposure, with damage susceptibility increasing under combined thermal fluctuations and mechanical loading. Additionally, the effects of moving load magnitude and speed on pavement damage were examined, showing higher damage accumulation at lower speeds and heavier loads. This research provides insights into pavement deterioration mechanisms, contributing to improved durability and maintenance strategies for asphalt pavements in salt environments.

## 1. Introduction

The use of de-icing salts is a common and economical strategy for ensuring safe road conditions in winter across many countries [1,2,3,4]. However, these chemicals can adversely affect asphalt pavements, resulting in permanent damage that undermines their durability and sustainability [5,6]. Previous studies have shown that chemical reactions initiated by de-icing salts not only accelerate the deterioration of pavement materials but also pose challenges to the long-term resilience of road infrastructure. Therefore, understanding how salt impacts asphalt performance is essential for developing strategies to enhance the longevity and sustainability of pavements.

The effects of salt on asphalt pavement can be analyzed at three main scales: micro-scale, meso-scale, and macro-scale. At the micro-scale, techniques such as infrared spectroscopy (FTIR) analysis and scanning electron microscopy (SEM) are used to observe changes in both the chemical composition and the morphological characteristics of asphalt before and after salt exposure [3]. These techniques can accurately analyze changes in the chemical composition of asphalt and the effects of salt-induced chemical reactions on its polymer chains. Furthermore, transmission electron microscopy (TEM) and atomic force microscopy (AFM) are employed to investigate the mechanisms of salt-induced damage to the microstructure of asphalt. These techniques enable the identification of changes in interfacial interactions between asphalt particles as well as between asphalt and fillers during salt erosion. Additionally, molecular dynamics (MD) simulations are utilized to investigate the erosion process induced by salts, revealing the molecular interactions and structural changes that occur during this process [7,8,9].

The meso-scale studies primarily focus on the fine asphalt matrix (FAM) and asphalt mixtures. Traditionally, experimental methods have been the principal approach for investigating the mechanical behavior of asphalt mixtures under the influence of salts. Semicircular bending (SCB) tests [10,11,12], indirect tensile (IDT) strength tests [13], and fatigue tests, have been used to measure the mechanical properties, and failure mechanisms of asphalt concrete under different conditions. Additionally, various laboratory tests that consider the effects of salt have been conducted to assess the damage to asphalt concrete [14,15]. These tests provide intuitive parameters and results to evaluate the performance of asphalt concrete and inform pavement construction practices. Furthermore, macro-scale experiments that simultaneously consider the effects of temperature and salt have been performed to investigate the deterioration of mechanical properties in asphalt concrete [14,16]. However, while experimental methods are important for understanding fundamental behaviors at a single scale, deriving the mechanisms by which environmental factors affect macro-scale pavement deterioration from these results is challenging. Therefore, integrating research across different scales is essential for a better understanding of the combined effects of salt and temperature on the long-term performance of asphalt materials.

Due to the limitations of experimental methods, numerical simulations have increasingly been employed in pavement research. Methods such as the discrete element method (DEM), finite element method (FEM), and finite difference method (FDM) have been successfully applied to simulate temperature fields and stress distributions within pavement structures [17,18,19,20,21,22,23]. These approaches facilitate detailed analyses at both micro and macro scales, contributing to a better understanding of the damage mechanisms of asphalt pavements. Additionally, numerical simulations offer the advantage of being able to model complex interactions and predict the long-term performance of materials. However, currently numerical studies mainly focus on the thermal–mechanical coupling effect on asphalt concrete [24,25,26] or focus on isolated factors by changing some specific parameters of the model, which may not fully capture the coupled interactions among salt diffusion, thermal effects, and mechanical loading.

**Figure 1:**
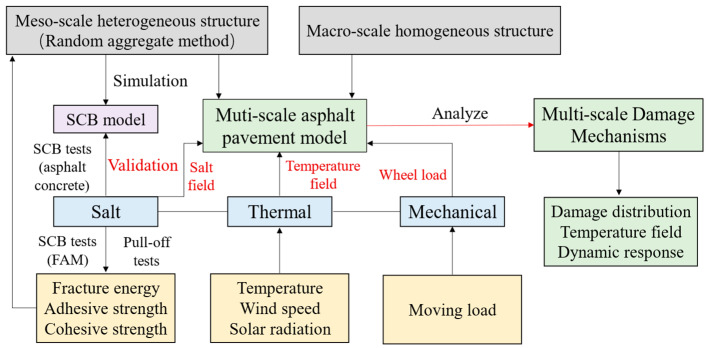
Flowchart of multi-scale modeling and damage mechanisms analysis.

Consequently, the main objective of this study is to investigate damage mechanisms of asphalt pavements under coupled salt–thermal–mechanical effects based on multi-scale modeling analysis. By systematically integrating experimental measurements with advanced numerical simulations, the research aims to provide fundamental insights into the complex environmental factors that influence asphalt material performance and structural durability. A multi-scale pavement model involving a local heterogeneous asphalt concrete course was developed. The damage and fracture of the asphalt concrete were simulated by embedding adhesive and cohesive elements. Various laboratory experiments were conducted to determine the material parameters for the asphalt concrete and multi-scale pavement FE model. The accuracy of the models was validated by three-point bending tests of asphalt concrete. Additionally, with the validated model, the damage mechanisms of pavement under coupled salt–thermal–mechanical effects were investigated in this study. The flowchart of this study is shown in Figure 1.

## 2. Theories

**Figure 2:**
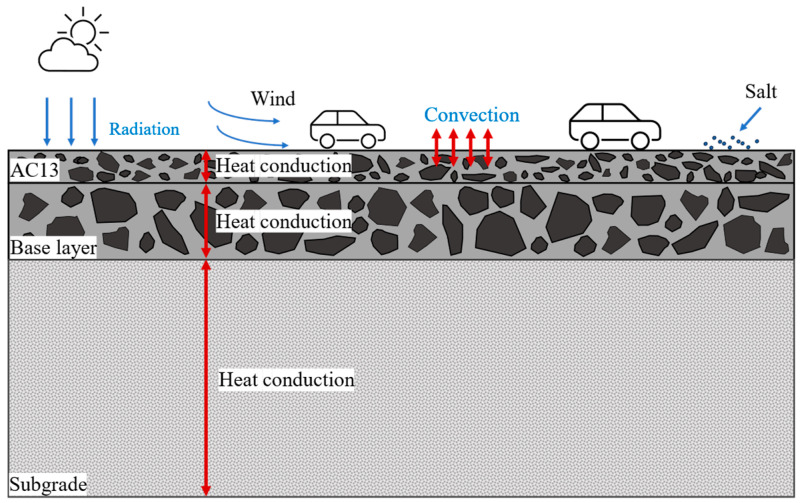
Schematic diagram of environmental and mechanical effects of pavement.

For a deeper understanding of the mechanisms affecting pavement performance, the penetration of salt ions, the thermal behavior of the pavement, and the material degradation process on pavement response were considered, as shown in Figure 2. Three theories were employed to investigate the damage mechanisms in this study.

### 2.1. Diffusion Theory

**Figure 3:**
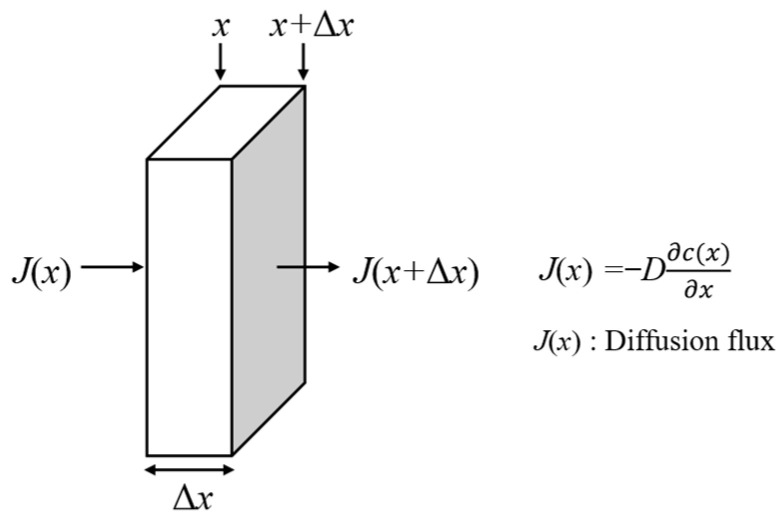
Schematic diagram of Fick’s second law.

During winter road de-icing operations, salt ions can penetrate the interior of asphalt pavement, causing deterioration of pavement materials. In this process, the non-stationary diffusion of salt ions can be characterized by Fick’s second law, as shown in Figure 3. Assuming that the diffusion coefficient is independent of concentration and only vertical diffusion is considered, the following equation can be used to characterize it:

(1) $$\frac{\partial c}{\partial t}=D\frac{\partial^{2}c}{\partial x^{2}}$$

where *c* is the concentration; *t* is time; *x* is the position coordinate, presenting the direction of diffusion; *D* is the diffusion coefficient. By setting the initial concentration and boundary conditions and using numerical methods for calculation, the concentration distribution of salt ions at different times can be obtained.

### 2.2. Damage Theory

**Figure 4:**
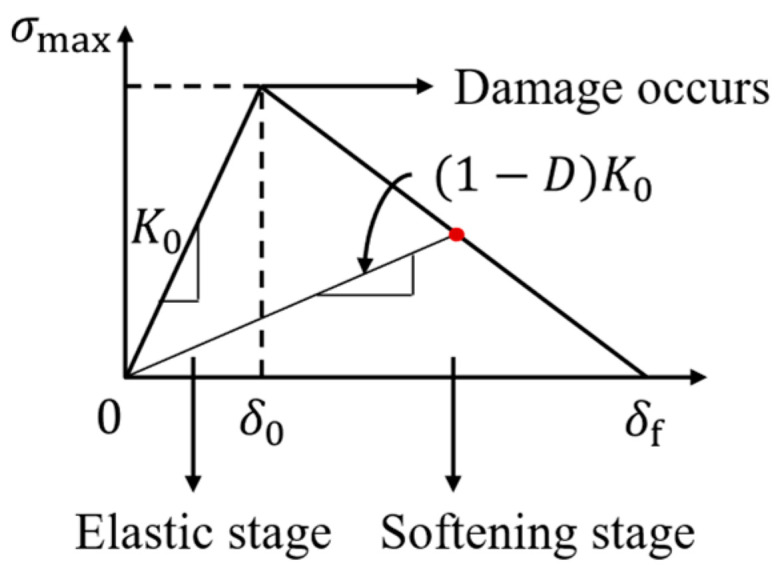
Schematic diagram of bilinear traction-separation law.

Due to the fracture strength of aggregates being much larger than that of the fine aggregate matrix (FAM) and the interface, cracks were commonly assumed that only appeared in the FAM and the FAM-aggregate interface in previous models [14,27]. In this study, cohesive elements were incorporated into FE models to simulate crack initiation and evolution in materials. To simulate the process of fracture in asphalt concrete, the Bilinear Traction-Separation Law was used to describe the behavior within the cohesive elements, as illustrated in Figure 4. This law defines the relationship between the traction force and the relative displacement of the crack surfaces.

Initially, when the relative displacement is less than the initial damage displacement *δ*_0_, a linear relationship exists between traction force and displacement, indicating the elastic stage of the cohesive element. As the crack displacement exceeds *δ*_0_, the material undergoes irreversible softening, resulting in a progressive reduction in stiffness. Ultimately, when the crack displacement surpasses the final displacement (*δ*_f_), the stiffness drops to zero, signifying complete material failure.

### 2.3. Heat Transfer Theory

The pavement temperature field is influenced by three primary modes of heat transfer: thermal radiation, thermal convection, and thermal conduction. In this study, based on existing research, the combined effects of these heat transfer modes were considered, and the following pavement heat equations, as the control equation for the pavement temperature field are shown below.

(1) Thermal Radiation

When solar radiation reaches the surface, it is not fully absorbed. The solar radiation that can enter the surface is shown in the Equation.

(2) $$Q_{\mathrm{in}}=(1-\tilde{\alpha })Q_{s}$$

where *Q*_in_ is the solar radiation entering the surface, W/m^2^; $\tilde{\alpha }$ is the surface reflectivity; *Q*_s_ is the solar radiation reaching the surface, W/m^2^.

The surface absorbs radiant heat under sunlight while also radiating heat to the surrounding environment. The radiation effect is typically influenced by the reflectivity and absorptivity of the surface material. According to the Stefan–Boltzmann law, the radiative heat flux density can be expressed as follows:

(3) $$Q_{\mathrm{out}}=\varepsilon \sigma (T^{4}-T_{\mathrm{ambient}}^{4})$$

where *Q*_out_ is the relative heat flux, W/m^2^; *ε* is the emissivity; *σ* is the Stefan-Boltzmann constant, 5.67 × 10^−8^ W/m^2^·K^4^ [20]; *T* is the pavement temperature, K; *T*_ambient_ is the ambient temperature, K.

(2) Thermal Convection

There is also convective heat transfer between the surface and the atmospheric environment, where the air carries away heat from the road surface through its flow. Thermal convection can be expressed using Newton’s law of cooling:

(4) $$q_{c}=h(T-T_{\mathrm{ambient}})$$

where *q*_c_ is the convective heat flux, W/m^2^; *h* is the convective heat transfer coefficient, W/m^2^·K, *h* = 5.6 + 4 × *v*_wind_ [20]; *T* is the pavement temperature, K; *T*_ambient_ is the ambient temperature, K.

(3) Thermal Conduction

Within the road structure, heat flow is transferred through conduction due to temperature gradients, which can be calculated using Fourier’s law:

(5) q=−k∇T

where *q* is the heat flux, W/m^2^; *k* is the thermal conductivity, W/mK; ∇*T* is the temperature gradient, K/m.

Therefore, the balance equation in this study is as follows:

(6) $$Q=\varepsilon \sigma (T^{4}-T_{\mathrm{ambient}}^{4})+h(T-T_{\mathrm{ambient}})-k\nabla T$$

## 3. Materials and Experiments

### 3.1. Experimental Research Program

A series of laboratory experiments was conducted to obtain the material parameters required for the FE model and to validate the model. The adhesive strength and cohesive strength of the asphalt concrete were determined through pull-off tests, while the fracture energy parameters of the FAM and the force-displacement curves of the asphalt concrete were obtained through SCB tests. The test details are shown in Table 1. Detailed methodological procedures are elaborated in Section 3.3 and Section 3.4.

### 3.2. Materials

In this study, asphalt concrete samples were prepared with basalt aggregates, mineral filler, and SBS-modified asphalt. The particle size of coarse aggregate in this study was 2.36–16 mm, and the FAM is a mixture of fine aggregate (particle size ≤ 2.36 mm), mineral filler, and SBS-modified asphalt. The gradation of aggregates used in the experiment is shown in Table 2. Moreover, the optimum binder content of asphalt concrete specimens was determined as 4.6% by total weight. The target air void content of asphalt concrete and FAM was 4%.

### 3.3. Pull-Off Test

The pull-off tests were conducted to measure the adhesive strength of the FAM-coarse aggregate interface, as well as the cohesive strength within the FAM. To investigate the adhesive and cohesive strengths of the FAM and coarse aggregate under different salt solution conditions, pull-off specimens were prepared and subjected to different experimental conditions. Given that salt intrusion is a long-term and complex process that is difficult to fully simulate real-world conditions, this study simplifies the model calculations and analysis by referencing previous studies [5,28]. Therefore, specimens were immersed in salt solutions with concentrations of 0, 3, 6, and 9%, at a temperature of 0 °C for durations of 0, 12, 24, 36, and 48 h. The fracture of asphalt concrete may occur in the following locations: (1) within the coarse aggregate; (2) at the interface between the coarse aggregate and the FAM; and (3) within the FAM itself. Generally, the fracture strength of the aggregates is significantly greater than that of the aggregate–FAM interface and the FAM [29,30,31]. Therefore, this study focuses exclusively on cases (2) and (3).

**Figure 5:**
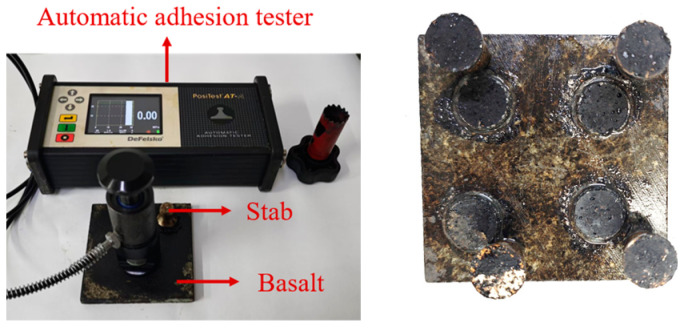
Automatic adhesion tester and specimen.

To quantify the adhesive strength at the aggregate–FAM interface (*σ*_FAM-aggregate_) and the cohesive strength within the FAM (*σ*_FAM_), an automatic adhesion tester (DeFelsko, Ogdensburg, NY, USA) was used to apply a controlled pull-off force to the specimens until failure occurred, as shown in Figure 5.

The steps for the pull-off test are as follows:

(1) The pull-off specimen was positioned within the sleeve of the pull-off apparatus to ensure stable installation, preventing any potential displacement during the testing procedure.
(2) The pull-off rate was set to 0.6 MPa/s. The testing apparatus was then to be activated, which would trigger the automatic commencement of the test, applying the specified pull-off force to the specimen at the predetermined rate.
(3) Upon completion of the test, the values displayed on the screen of the testing apparatus were recorded. Three parallel tests of each group were conducted to ensure statistical validity.

Following the pull-off test, the specimen is expected to fracture, potentially exhibiting one of four distinct fracture types: (a) adhesive failure at the interface of metal stab and FAM, where the fracture path follows the interface between the metal stab and FAM; (b) adhesive failure at the coarse aggregate–FAM, with the fracture path along the interface between the basalt aggregate and the FAM layer; (c) cohesive failure within the FAM, characterized by the fracture path traversing through the FAM layer; and (d) mixed failure, which incorporates a combination of the aforementioned failure types. A digital image processing technique was used to analyze the fracture surfaces, distinguishing between adhesive and cohesive failures [32,33]. Statistical analysis and calculations of the different fracture situations will be performed based on existing methodologies. The adhesive strength (*σ*_FAM-aggregate_) and cohesive strength (*σ*_FAM_) will be obtained from multiple tests for each specimen, and the average values will be calculated to serve as fracture parameters for the finite element model.

**Figure 6:**
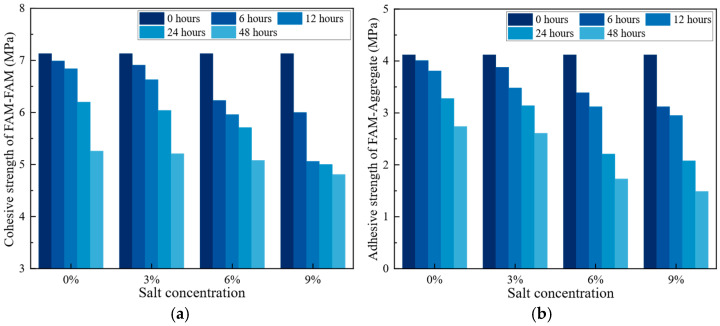

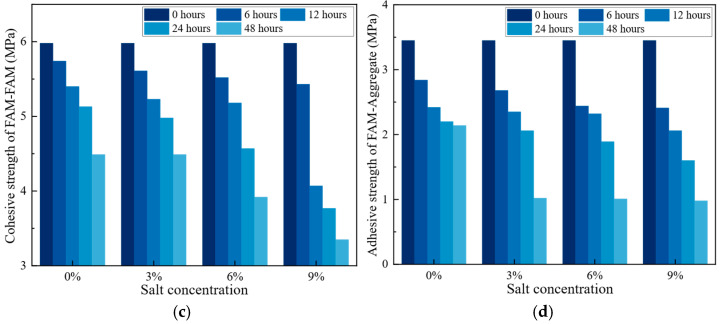
Cohesive strength and adhesive strength under different temperatures and salt concentrations. (**a**) cohesive strength (FAM-FAM) at −10 °C; (**b**) adhesive strength (FAM-aggregate) at −10 °C; (**c**) cohesive strength (FAM-FAM) at 0 °C; (**d**) adhesive strength (FAM-aggregate) at 0 °C.

The strength parameters of the material were tested and calculated under different salt solution concentrations and soaking durations. Additionally, conditions at −10 °C and 0 °C were tested. To avoid the interference of freezing and thawing effects on the results, before conducting the pull-out test and SCB test at −10 °C and 0 °C environments, the specimens were soaked in a 0 °C salt solution. The obtained parameters were used in the subsequent FE models to simulate the behavior of the pavement materials. Each set of specimens consists of three groups, and the average values were computed. The results are shown in Figure 6.

As illustrated in Figure 6, both cohesive and adhesive strength values declined with increasing salt concentration and immersion time. At −10 °C, the cohesive strength after 48 h of treatment decreased by 26% to 32.5% compared to the control group, while the adhesive strength decreased by 33.5% to 63.8%. Additionally, with the same treatment duration, the cohesive strength decreased by 8% to 26%, and the adhesive strength decreased by 22.2% to 45.6%. Therefore, the treatment duration has a more significant effect on both cohesive and adhesive strength. At 0 °C, the cohesive strength after 48 h of treatment decreased by 24.9% to 44.0% compared to the control group, while the adhesive strength decreased by 37.9% to 71.6%. Additionally, with the same treatment duration, the cohesive strength decreased by 5.4% to 26.5%, and the adhesive strength decreased by 14.9% to 54.2%. It is important to emphasize that even in the absence of salt, wet treatment (immersion in a 0% salt concentration solution) leads to a reduction in the strength of the FAM–aggregate and FAM–FAM interfaces, which is consistent with previous results [34].

These reductions in cohesive and adhesive strengths suggest that prolonged exposure to salt solutions at low temperatures may compromise the internal bonding within the FAM. This decline could potentially lead to increased susceptibility to cracking and structural failure. Furthermore, the diminished adhesive strength at the FAM-aggregate interface could result in reduced overall durability and performance of the pavement, increasing the likelihood of surface damage and necessitating more frequent maintenance.

### 3.4. SCB Test

A series of SCB specimens 150 mm in diameter, 75 mm in height, and 30 mm in thickness were prepared for obtaining the fracture parameters of FAM and asphalt concrete. These samples were cut from the cylindrical specimens of FAM and asphalt concrete 150 mm in diameter and 150 mm in height, which were prepared with the Superpave Gyratory Compactor (SGC). Additionally, an incision with 2 mm in width and 15 mm in length was made at the bottom center of each semi-circular specimen. To simulate the deicing salt environment, SCB specimens were placed in a 0 °C environment and soaked in deicing salt solutions with concentrations of 0, 3, 6, and 9%. The soaking durations were 0, 6, 12, 24, and 48 h. After treatment, the surface moisture of the specimens was wiped off, and SCB tests were conducted at −10 °C or 0 °C. Each test condition included three parallel specimens to ensure the reliability of the results.

**Figure 7:**
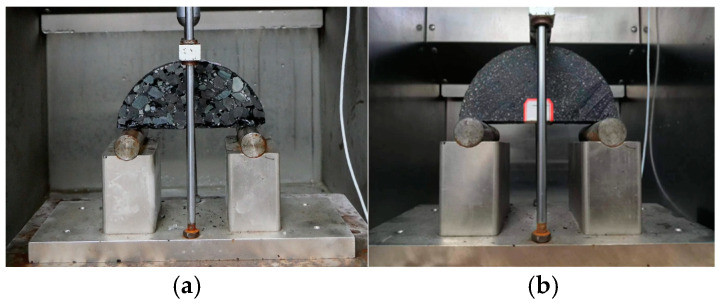
Test equipment and samples: (**a**) Three-point bending test of asphalt concrete, (**b**) SCB test of FAM.

In the experiment, a universal testing machine (UTM-250, IPC Global, Boronia, VIC, Australia) equipped with an environmental chamber was used to control the temperature, as shown in Figure 7. The specimens were placed on two metal supports in the environmental chamber without any constraints; the distance between the two bottom supports was 120 mm, and the vertical loading rate was set at 5 mm/min. A waiting period of over 4 h was required to ensure that the temperature within the specimens was uniform. Force-displacement curves were then obtained to determine the fracture properties of the FAM and asphalt concrete.

**Figure 8:**
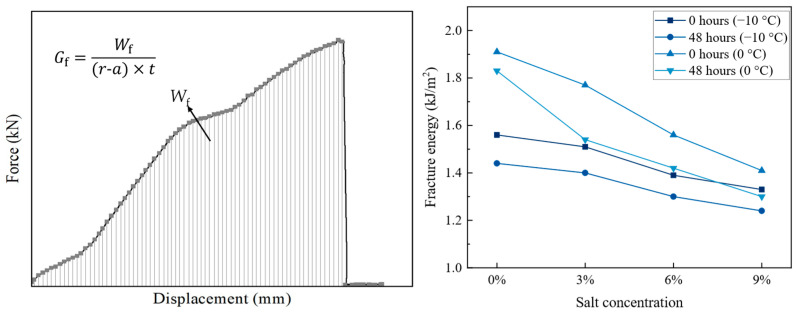
Fracture energy of FAM.

In this study, the FAM was considered as a continuous matrix within the asphalt concrete, which could influence the overall fracture performance. Previous research has demonstrated that fracture energy (*G*_f_) is a crucial indicator for evaluating fracture performance [5]. The specific calculation methodology is presented as Equation (7) [35]:

(7) $$G_{f}=\frac{W_{f}}{(r-a)\times t}$$

where *W*_f_ is the fracture work, defined as the area enclosed by the load–displacement curve and the coordinate axes, mJ; *r* is the radius of the specimen of SCB, mm; *a* is the length of the notch, mm; *t* is the thickness of the specimen, mm. The fracture energy values of the FAM under different salt concentrations were calculated using Equation (7), and the results are presented in Figure 8.

The lines in Figure 8 showed that the fracture energy was affected by both the salt concentration, temperature, and treatment time. The fracture energy at −10 °C is lower than that at 0 °C. In addition, the fracture energy that decreases at 0 °C is larger than that at −10 °C, with values of 4.2~13%. This reduction in fracture energy at 0 °C may suggest an increased risk of crack propagation in asphalt concrete under these conditions. Existing research has demonstrated that lower fracture energy corresponds to a higher rate of crack growth, indicating that asphalt pavements exposed to similar low-temperature environments could experience accelerated damage over time [36,37].

## 4. Model Development

Two types of models were developed in this study to investigate the damage of asphalt concrete pavement under coupled salt–thermal–mechanical effects. One is the heterogeneous SCB model to study the fracture mechanism of asphalt concrete and to validate the accuracy of the heterogeneous structural model, the other is the multiscale pavement model. The multi-scale pavement model includes heterogeneous and homogeneous pavement layers, homogeneous UBG (unbound granular) layers, and base layers. By analyzing meso-scale damage and macro-scale heat transfer, the damage mechanisms of asphalt pavements under salt–thermal–mechanical coupling were investigated. At the macro-scale, the vertical transfer of pavement temperature was considered. The mechanical model at the meso-scale consists of three components: salt transport, the deteriorative effects of salt on the *σ*_FAM-aggregate_ and *σ*_FAM_, and damage under the combined action of salt and mechanical loads. In this study, cracks can occur within the FAM (cohesive damage) or along the interface between the aggregate and FAM (adhesive damage).

### 4.1. Heterogeneous SCB Model

**Figure 9:**
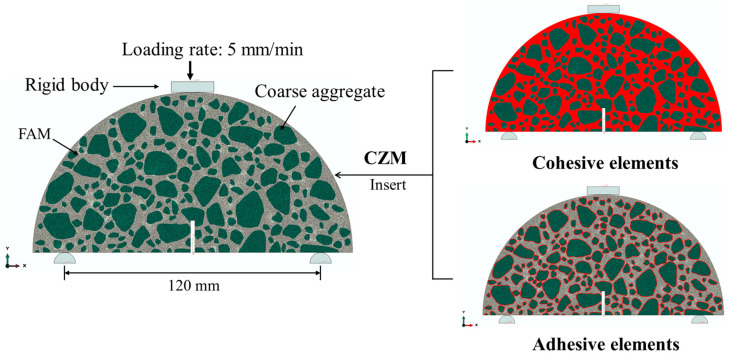
The schematic of the asphalt concrete FE model.

In this study, a heterogeneous asphalt concrete model was developed using digital image processing (DIP) techniques and the random aggregate method. Aggregates with a particle size greater than 2.36 mm were classified as coarse aggregates, while the Fine Asphalt Mixture (FAM) was defined as a combination of fine aggregates (particle size ≤ 2.36 mm), mineral powder, and asphalt. The Aggregate Image System (AIMS) was used to obtain 2D images of the coarse aggregates [38], and the gradation of coarse aggregates and the size of the models were consistent with those of the specimens in the SCB experiments. Based on the random aggregate method, all coarse aggregates were randomly placed in a designated area. Then, the FAM phase was determined through Boolean operations. Additionally, three rigid bodies were developed to simulate the SCB test setup. The horizontal distance between the centers of the two bottom rigid bodies under the model was 120 mm, consistent with the dimensions of the SCB specimens in the experimental tests. The schematic of the SCB model is shown in Figure 9.

In the heterogeneous SCB models, both the coarse aggregates and the FAM were meshed using 3-node linear elements. Cohesive elements with zero thickness were inserted at the FAM–aggregate interface and within the FAM in the meshed meso-scale model to represent damage evolution and crack propagation. Specifically, the cohesive elements at the FAM–aggregate interface were employed to replicate interfacial adhesive behavior, while those within the FAM represented internal cohesive behavior [31,39], as shown in Figure 8. A vertical displacement of 5 mm/min was applied to the top rigid body to simulate the SCB test process.

The composition of the asphalt concrete was established based on findings from previous studies, which also provided the basis for deriving the dynamic modulus used in the models. The basic viscoelastic material parameters, including the elastic modulus and Poisson’s ratio, were consistent with those of the FAM [40]. The detailed parameters of the models are presented in Table 3 and Table 4.

### 4.2. Multi-Scale FE Pavement Model

In this study, a multi-scale asphalt pavement model with a length of 3000 mm and a total thickness of 3000 mm was developed to minimize boundary effects. The model consisted of three layers with distinct materials: an asphalt concrete layer (AC-13), a cement-stabilized gravel base layer (Base layer), and a subgrade layer [41]. Except for the asphalt concrete layer, the other layers were considered homogeneous.

**Figure 10:**
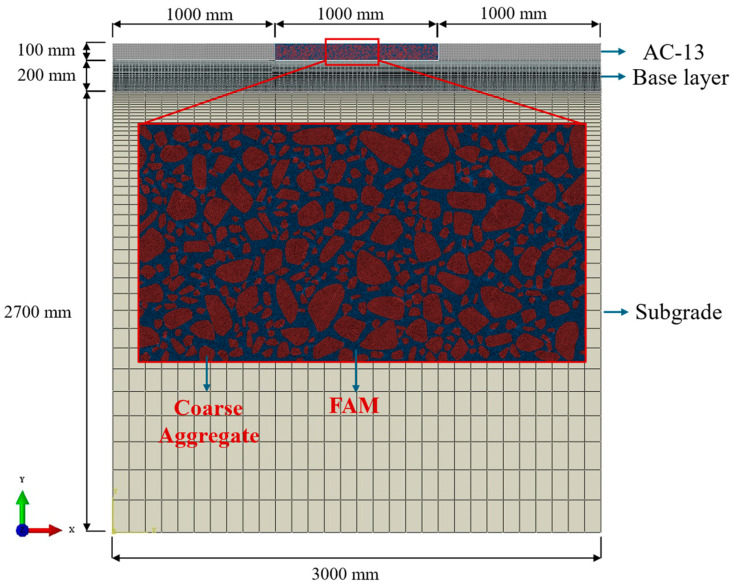
Multi-scale FE pavement model.

To reduce computational costs, the asphalt concrete layer was divided into heterogeneous and homogeneous sections. The parameters of the pavement model for the heterogeneous portion were maintained consistently with the laboratory tests. Detailed information on each layer of the pavement model is shown in Figure 10.

Under traffic loads, asphalt concrete exhibits time-dependent stress and strain behavior. To accurately simulate the asphalt pavement’s response to dynamic loads in this multi-scale pavement model, viscoelastic parameters were assigned to both the heterogeneous FAM and the homogeneous sections of the AC-13 layer. In addition, the other layers were considered as linear elastic materials in this study. The detailed parameters of the models are shown in Table 4 and Table 5 [5].

In the multi-scale pavement model, all displacements at the bottom were constrained to zero, while vertical displacements at the lateral boundaries were permitted. The heterogeneous portion of the pavement model was meshed using 3-node linear elements, whereas the remaining homogeneous sections were discretized using 4-node bilinear elements. In this study, finite element analysis was conducted using ABAQUS/Standard software (2021) with a dynamic implicit analysis method. The interface between the asphalt surface layer and base layer was modeled using a tied contact approach to ensure precise stress transmission and mechanical continuity.

To facilitate the subsequent analysis and computation of pavement temperature fields, the following fundamental assumptions were established in this study:

(1) Except for the heterogeneous pavement structural regions, all other structural layers were assumed to be homogeneous, continuous, and isotropic.
(2) The materials between the pavement layers were assumed to be tightly bonded, ensuring continuity of temperature and heat flow at the interfaces.
(3) The lateral variation in the pavement temperature field was disregarded under the assumption that heat flow occurs solely in the vertical direction of the pavement.

## 5. Results and Discussion

### 5.1. Model Validation

**Figure 11:**
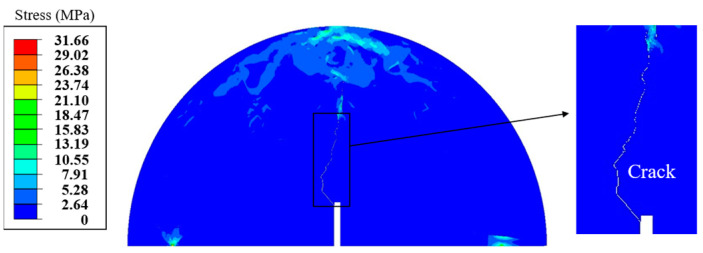
Schematic diagram of the damaged SCB model.

**Figure 12:**
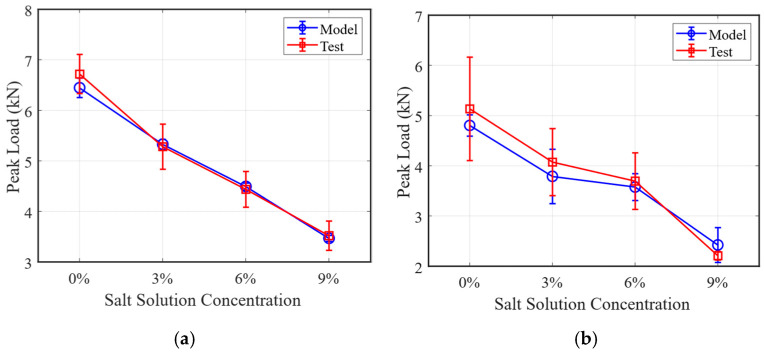
Comparison of the peak loads of FE models and tests at: (**a**) −10 °C, (**b**) 0 °C.

To evaluate the accuracy of the computational models described above, three heterogeneous SCB asphalt concrete models with different aggregate distributions were developed. Peak load and fracture energy values for the asphalt concrete models under various conditions were obtained through the simulation of SCB tests. Moreover, the parameters for these conditions were kept consistent with those used in laboratory experiments. The schematic diagram of the damaged SCB model is shown in Figure 11. Based on the provided SCB test results and finite element simulations conducted at different temperatures (0 °C and −10 °C, treatment for 48 h), the peak load values of asphalt concrete specimens were compared under varying salt solution concentrations, as shown in Figure 12.

As shown in Figure 12, both experimental and simulation results indicate a consistent reduction in the peak load of asphalt concrete with increasing salt solution concentrations. Moreover, the computational values from the models generally fall within the range of the experimental results, suggesting that the overall accuracy of the model is acceptable. Specifically, at −10 °C, the peak load decreased from approximately 6.6 Kn at 0% salt concentration to around 3.5 Kn at 9% concentration. In contrast, at 0 °C, the peak load declined from around 5 Kn to 2.4 Kn as the salt concentration increased. Additionally, the peak load was 21.7% lower in the absence of salt after wet–dry cycles compared to that of the control group. Moreover, when treated with salt solutions, the peak load decreased by 37.4% compared to the control group (salt concentration = 0%).

### 5.2. Analysis of Salt Diffusion and Temperature Field of Pavement

**Figure 13:**
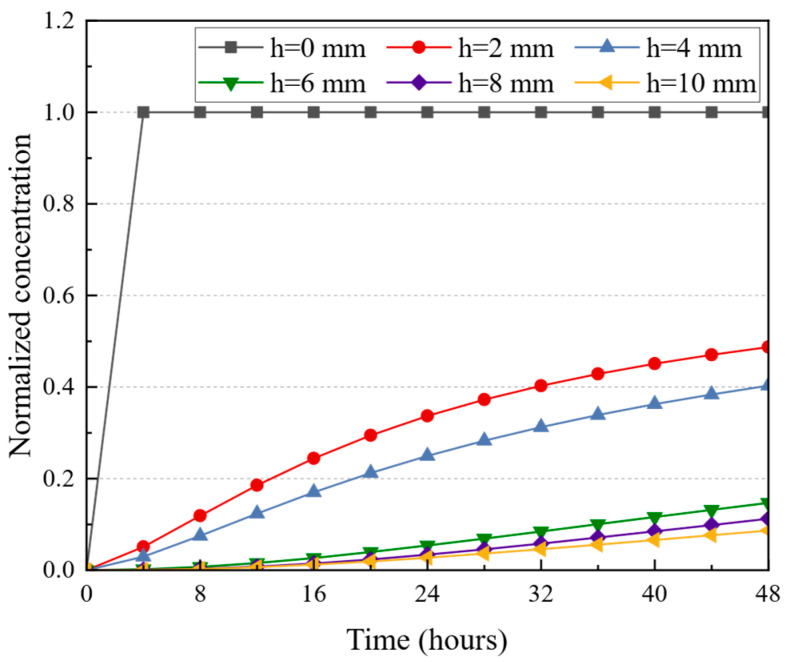
Changes in normalized concentration at different depths of pavement.

In the investigation of salt damage to dense-graded asphalt pavement, salt diffusion is a critical factor. Salt ions diffuse into the pavement interior through water infiltration, yet they are difficult to reach deeper layers within a short time. Therefore, analyzing salt diffusion is essential. The salt diffusion in heterogeneous pavement structures was analyzed in this section, specifically calculating salt migration through water-mediated transport over 48 h. The computational model defined boundary conditions where the top surface had a salt concentration of 1 (representing fully saturated conditions) and the bottom surface had a concentration of 0 (indicating a completely dry state). Normalized concentration was employed to represent moisture (salt) content, with values ranging from 0 and 1. The results are shown in Figure 13.

Figure 13 illustrates a pronounced disparity in normalized concentration changes across various depths of the asphalt pavement in 48 h. Initially, the road surface (h = 0 mm) exhibits a rapid increase in normalized concentration, indicating a high rate of salt ion penetration due to direct exposure to the saturated surface conditions. In contrast, deeper layers (h = 8 mm and h = 10 mm) demonstrate a markedly diminished rate of concentration change, suggesting a substantial reduction in the efficacy of water-mediated transport as depth increases. These findings illustrate the heterogeneity of the pavement structure, with upper layers nearing saturation while deeper layers remain relatively unsaturated.

**Figure 14:**
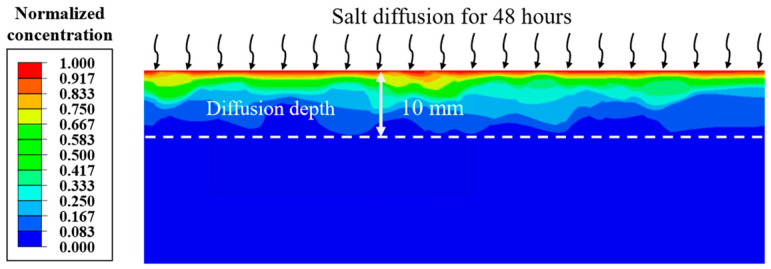
Salt concentration distribution of asphalt pavement at 48 h.

Moreover, Figure 14 illustrates the distribution of salt within the pavement structure. The results reveal that after 48 h, salt ions approximately diffuse to a depth of 10 mm from the pavement surface. This observation aligns with findings from previous studies, where similar diffusion depths were reported, thereby supporting the reliability of the results presented in this study [42]. Moreover, due to differential diffusion coefficients between aggregate and FAM, the internal salt distribution exhibits notably non-uniform characteristics. This is because the coarse aggregates and FAM create a complex diffusion pathway due to their different diffusion coefficients. These distinct diffusion coefficients cause salt ions to interact and migrate differently within each material component, leading to localized concentration variations across the pavement structure.

In addition to salt diffusion, the pavement temperature experiences periodic fluctuations due to solar radiation and diurnal cycles. Variations in solar radiation cause the atmospheric temperature to fluctuate periodically. Typically, the daily minimum temperature occurs before dawn, while the maximum temperature is reached approximately two hours after peak solar radiation. Consequently, it takes less than 10 h for the temperature to rise from the minimum to the maximum, while it takes over 14 h to drop back to the minimum. Since a single sine function cannot adequately capture this complex temperature change, a linear combination of two sine functions is employed for modeling [41].

(8) $$T=T_{a}+T_{m}[0.96\sin\omega (t-t_{0})+0.14\sin2\omega (t-t_{0})]$$

where *T*_a_ is the daily average temperature, *T*_a_ = 1/2(*T*_max_ + *T*_min_), °C; *T*_max_ and *T*_min_ are the daily maximum and minimum temperatures, respectively; *T*_m_ is the daily temperature amplitude, *T*_m_ = 1/2(*T*_max_-*T*_min_), °C; *ω* is the angular frequency, *ω* = 2π/24, rad; the phase shift *t*_0_ is 9, accounting for the time difference between peak solar radiation and maximum temperature. In calculations, *t* is expressed in hours.

Solar radiation is represented using a fitting method from the existing study [41]:

(9) $$q(t)=q_{0}\cos m\omega (t-12),(12-\frac{c}{2}\leq t\leq 12+\frac{c}{2})$$

where *q*_0_ is the maximum radiation at noon, *q*_0_ = 0.131 M*q*, *m* = 12/c; *c* is the actual effective sunshine duration, hour. *Q* is the total daily solar radiation, J/m^2^.

**Figure 15:**
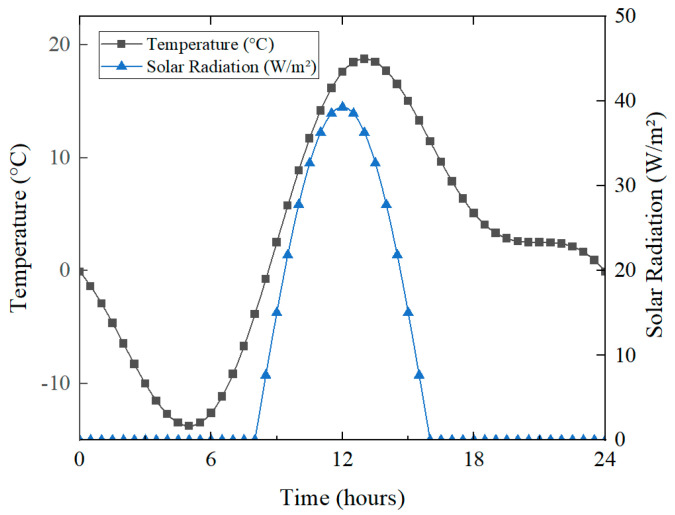
Temperature and solar radiation change over 24 h.

Under winter de-icing conditions, pronounced temperature variations can induce significant thermal stress and potential pavement damage. Specifically, this study considers a scenario with *T*_max_ = 15 °C and *T*_min_ = −10 °C, analyzing the daily temperature fluctuations and solar radiation characteristics using the proposed fitting method. The results are illustrated in Figure 15.

**Figure 16:**
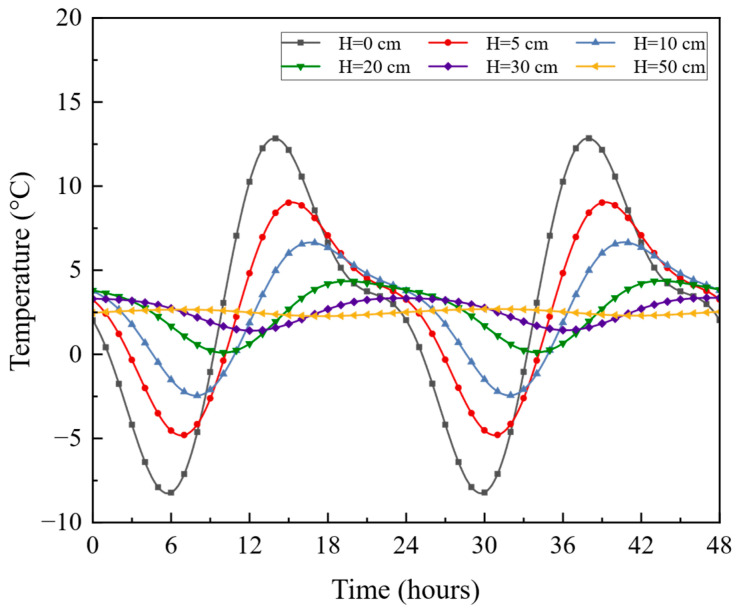
Pavement temperature values in 48 h.

To ensure stable cyclic variations in the pavement temperature field, this study utilized a finite element model to simulate five continuous daily temperature cycles, as illustrated in Figure 15. The temperature distribution at various pavement depths during the last 48 h is shown in Figure 16.

As shown in Figure 16, the temperature fluctuation range of the pavement surface (H = 0 cm) was the largest, which means the pavement surface was affected most by the weather conditions. At the same time, with the depth increasing, the temperature was gradually stable. At a distance of 50 cm from the pavement surface, the temperature values changed slightly. Given these observations, it is recommended to consider the implementation of thermal insulation layers within the pavement structure to help mitigate temperature fluctuations at the surface. Enhancing the temperature stability of the upper layers could reduce thermal stress, thereby extending the pavement’s lifespan and improving overall performance. Additionally, using materials with better thermal properties may further enhance the pavement’s resilience to temperature variations, promoting a more durable infrastructure in areas with significant temperature changes.

### 5.3. Responses to Coupled Salt–Thermal–Mechanical Effects

Based on the results from Section 5.2, de-icing salt primarily diffused within the shallow surface layer in a short time, with limited influence on deeper pavement regions. Consequently, the multi-scale pavement model focuses on the structural response from the surface to a depth of 10 mm. To improve computational efficiency, the cohesive element damage parameters for this layer were set to values obtained from experimental measurements conducted at 48 h.

**Figure 17:**
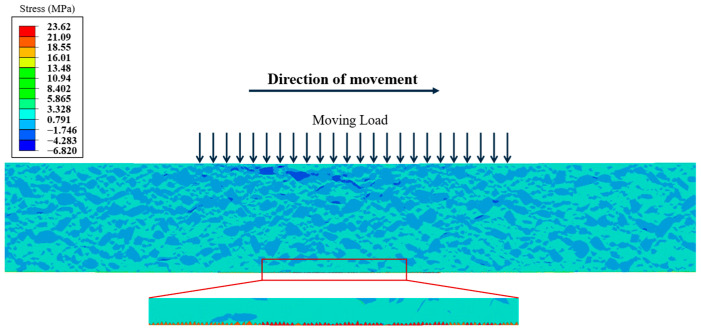
Distribution of stress under a moving load.

As shown in Figure 17, within the entire AC-13 layer (H ≤ 10 cm), the temperature variation within one day was 20 °C. The inherent differences in thermal expansion coefficients between coarse aggregate and FAM inevitably generate internal thermal stresses. Therefore, the temperature at the 14th hour was used as the initial temperature, and the temperature field at the 7th hour of the following day was applied as the boundary condition for computational analysis. Figure 17 shows the stress distribution inside the AC-13 layer when a moving load travels to a certain position.

The figure illustrates that within the entire asphalt layer, the internal structure near the position of the moving load primarily exhibits a compressive and tensile characteristic, with the maximum tensile stress occurring at the bottom of the layer. Additionally, in the area near the surface, temperature stress can be observed when the moving load is not present. This is primarily due to thermal expansion and contraction resulting from environmental temperature changes affecting the surface. Since the thermal expansion coefficient of the FAM is higher than that of the coarse aggregate, tensile stresses develop within the FAM as the temperature decreases.

### 5.4. Effects of Salt

**Figure 18:**
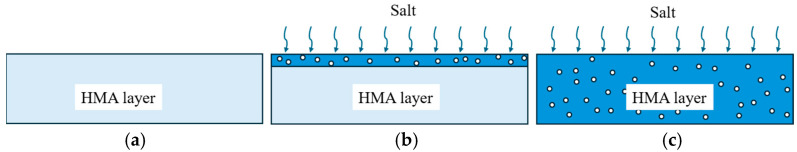
Stages of salt diffusion in asphalt pavement: (**a**) none; (**b**) shallow penetration; (**c**) full-depth penetration to the AC-13 base.

Based on the analysis in Section 5.2, it is evident that salt ions have difficulty diffusing deeply into the pavement in a short period (Figure 18b). To further investigate the influence of salt on the dynamic response of asphalt pavements, analyses were conducted under two conditions: one without salt (Figure 18a) and the other with salt diffusion reaching the bottom of the AC-13 layer (Figure 18c). Additionally, previous studies have shown that moisture damage affects the elastic modules of asphalt concrete; therefore, the elastic modulus of FAM was assumed to be 90% of the original elastic modulus.

**Figure 19:**
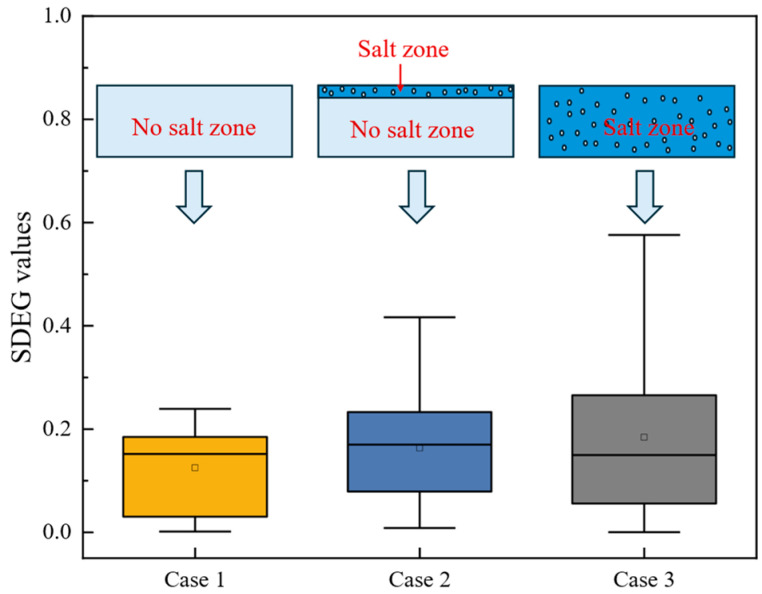
SDEG values under different salt diffusion conditions.

The pavement temperature field and moving load (40 km/h, standard load) were considered in the model, and the SDEG values for three different conditions were extracted and are presented in Figure 19. To enhance the visualization and interpretability of the model calculation results, box plots were employed, which offer a more comprehensive representation of data distribution and statistical characteristics.

As shown in Figure 19, the SDEG values increase with greater salt diffusion depth. In the case without salt influence (Case 1), the average SDEG is 0.1250, while salt diffuses to a depth of 10 mm in the pavement (Case 2), it rises to 0.1632. When salt completely infiltrates the AC-13 layer (Case 3), the average further increases to 0.1843, although the median slightly decreases to 0.1495. This decrease in median indicates that, although more cohesive elements have sustained damage, the severity of these damages is relatively minor, resulting in a lower median value despite the increasing average SDEG.

**Figure 20:**
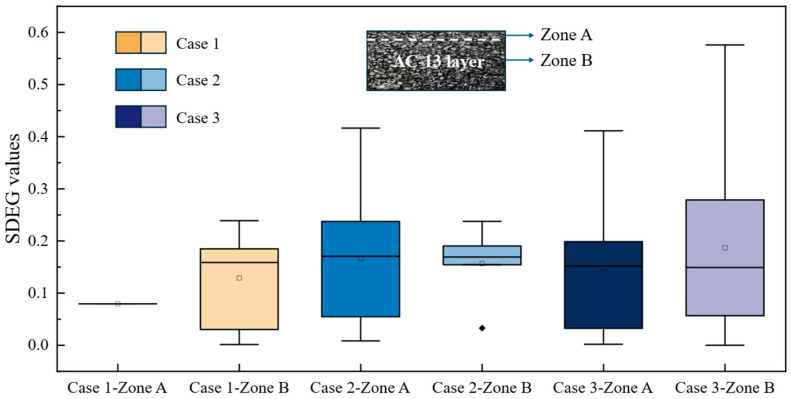
SDEG values under different salt diffusion conditions and different locations.

In addition, to compare the effect of salt on the damage of elements in different parts of the AC-13 layer, the structural layers under three conditions were divided into two regions, A and B. The results are shown in Figure 20. Figure 20 illustrates the SDEG values under different salt diffusion conditions. As shown in the figure, in Case 1, the cohesive elements in Zone A exhibit almost no damage, with damage primarily concentrated in Zone B. To be more specific, the cohesive elements in Zone A exhibit minimal damage, with median SDEG values close to 0.05, while Zone B shows significantly higher damage, with median values around 0.20. This substantial difference highlights the non-uniform stress distribution within the AC-13 layer. This is because, during significant temperature drops, the coefficient of thermal contraction of the FAM is greater than that of the coarse aggregate, causing the FAM to predominantly bear tensile stress. Additionally, the AC-13 layer experiences a state of compression in the upper layer and tensile stress in the lower layer under a moving load. Under the influence of thermal stress, the tensile stress in the lower layer increases, further causing damage to the structure.

Conversely, in Case 3, the cohesive elements in Zone A also experience damage due to temperature fluctuations and moving loads, with the damage degree of the cohesive elements at the bottom being significantly higher than that of Zone A. This indicates that the presence of salt reduces the structural load-bearing capacity. In Case 2, the damage level of the cohesive elements in Zone A exceeds that of Zone B. This result is attributable not only to stress factors but also to the reduction in cohesive strength caused by salt. The results in Figure 17 indicate that the deterioration of materials due to salt action is more pronounced than the effects caused solely by moving loads.

### 5.5. Effects of Moving Load

To investigate the effects of moving loads on the damage of asphalt pavements in salt environments, two distinct vertical loads were applied to the multiscale finite element model shown in Figure 10. The first load was a standard load of 50 kN, while the second was a heavy load of 100 kN. Assuming a contact pressure of 0.9 MPa, the moving load was simplified to a line load with an equivalent circular diameter in the 2D model [27,29]. The application of the moving load was implemented using a user-defined DLOAD subroutine in ABAQUS. Initially, the starting position, length, and speed of the wheel load were defined. Subsequently, the current coordinates of the load’s sides were calculated based on the given time. Finally, the subroutine determined the load value by assessing whether the current spatial coordinates of the analysis points fell within the dynamically updated load boundaries.

**Figure 21:**
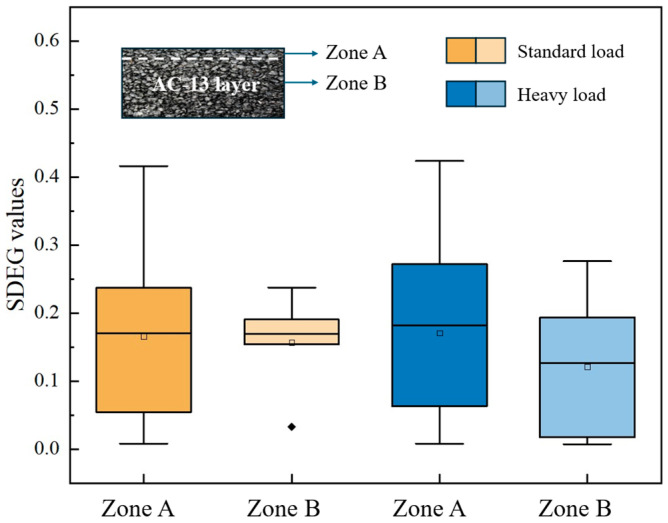
SDEG values under different moving loads.

Figure 21 demonstrates that the SDEG values for damaged cohesive elements under both standard and heavy loads exhibit similar effects on the upper section of the AC-13 layer. However, in the lower section of the layer, where there is no influence of salt, the maximum SDEG values of cohesive elements under heavy load increase by 16% compared to those under standard load. This increase suggests that heavy loads can lead to more significant localized damage in the absence of salt, which may weaken the cohesive elements further. Interestingly, the average SDEG value under heavy load is lower than that under standard load. This anomaly occurs because more cohesive elements sustain damage under heavy loads that do not experience damage under standard loads, leading to a situation where these zero values were not considered in the average calculation.

**Figure 22:**
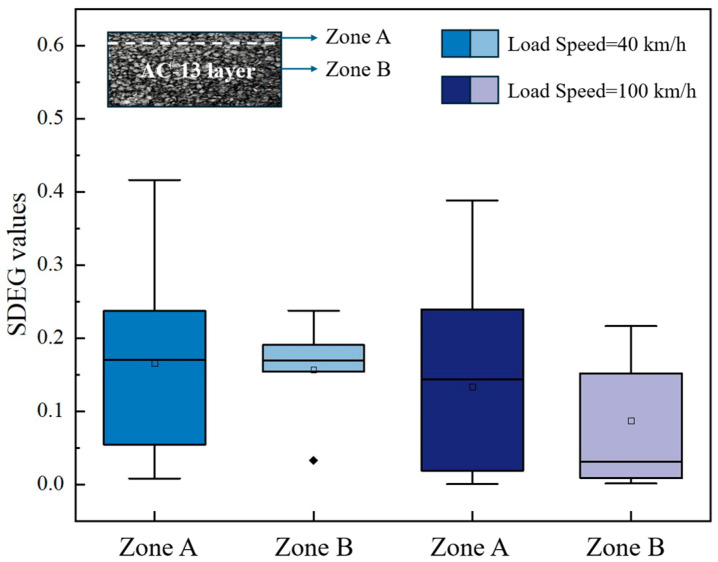
SDEG values under different moving load speeds.

Asphalt concrete is recognized as a viscoelastic material, with its dynamic response significantly influenced by the speed of the applied moving load. Therefore, two different speeds (40 km/h and 100 km/h) were considered in this study to study the effect of high and low speeds. When moving loads with different speeds acted at the same location as the model, the SDEG values of cohesive elements were obtained and are shown in Figure 22.

Figure 22 illustrates that the SDEG values under a moving load of 40 km/h are significantly higher than those observed under a 100 km/h load. This discrepancy can be attributed to the longer duration of stress application that occurs at lower speeds. When vehicles move at 40 km/h, they exert stress on the pavement for an extended period, resulting in greater stress accumulation within the material. This prolonged exposure allows more time for deformation and increases the likelihood of failure, ultimately leading to more extensive damage to the cohesive elements of the pavement structure.

Moreover, the lower speed of 40 km/h correlates with a reduced frequency of load application, enabling more substantial damage to accumulate between successive load impacts. In contrast, at 100 km/h, the increased frequency of load applications reduces the time available for stress relaxation within the material. Consequently, this finding suggests that maintenance strategies should not solely concentrate on high-speed traffic conditions but also consider the cumulative damage potential associated with lower speeds.

## 6. Conclusions

In this study, a multi-scale pavement FE model was developed to investigate the fracture mechanism of asphalt concrete and evaluate the damage of asphalt pavement under wheel load in a salt environment. CZM was used to simulate the fracture within the asphalt concrete model, and the SDEG value was employed to evaluate raveling susceptibility. The accuracy of the model was validated by laboratory experiments. Additionally, the damage mechanisms of pavement under coupled salt–thermal–mechanical effects were investigated in this study. The main findings of this study can be summarized as follows:

(1) According to the laboratory tests, at −10 °C, cohesive strength dropped by 26% to 32.5% and adhesive strength by 33.5% to 63.8% after 48 h. At 0 °C, cohesive strength decreased by 24.9% to 44.0% and adhesive strength by 37.9% to 71.6%. Additionally, fracture energy at 0 °C decreased by 4.2% to 13% compared to −10 °C.
(2) Salt ions diffused approximately 10 mm into the pavement after 48 h. The non-uniform distribution of salt within the pavement structure was attributed to the different diffusion coefficients of the aggregate and the FAM.
(3) Maximum tensile stress occurred at the bottom of the asphalt layer, with compressive and tensile characteristics exhibited near the moving load’s path. Temperature-related stresses manifested in the surface area even without the moving load, primarily driven by thermal expansion and contraction due to environmental fluctuations.
(4) The presence of salt significantly reduces the load-bearing capacity of the upper layer, resulting in greater damage to cohesive elements compared to the lower layer. Additionally, the detrimental effects of salt on material deterioration in the upper layer are more pronounced than those caused solely by the stresses from moving loads. It is recommended to implement measures to mitigate salt exposure on the upper layer of pavements.
(5) Lower moving speeds lead to increased damage in cohesive elements due to longer stress application times and lower load frequencies, resulting in greater stress accumulation compared to higher speeds.

To further confirm the reliability and applicability of the multi-scale asphalt concrete model developed in this research, it is recommended to incorporate additional validation methods, including field tests and evaluations of other mechanical properties.

## Figures and Tables

**Table 1:** Experimental research program information.

Type of Tests		Salt Concentration	Temperature	Immersion Duration	Sample Number
Pull-off test (FAM)		0, 3, 6, 9%	−10, 0 °C	0, 12, 24, 36, 48 h	120
SCB test	FAM	0, 3, 6, 9%	−10, 0 °C	0, 12, 24, 36, 48 h	120
	Asphalt concrete	0, 3, 6, 9%	−10, 0 °C	0, 48 h	48

**Table 2:** Aggregate gradations of asphalt concrete and FAM.

Sieve Size (mm)		16	13.2	9.5	4.75	2.36	1.18	0.6	0.3	0.15	0.075
Passing percentage (%)	Asphalt concrete	100	94.7	75.3	57.7	37.6	25.2	15.7	9.3	6.7	5.0
	FAM	100	100	100	100	100	67.0	41.9	24.8	17.7	13.3

**Table 3:** Parameters for materials in the heterogeneous model [40].

Type of Material	Elastic Modulus (MPa)	Poisson’s Ratio
Aggregate	80,000	0.20
FAM	805	0.25
CZM	805	0.25

**Table 4:** Parameters for the Prony series of FAM and asphalt concrete [26].

Temperature (°C)	*i*	Relaxation Time, *ρ_i_* (s)	Relaxation Strength, *E_i_* (MPa) FAM	Relaxation Strength, *E_i_* (MPa) Asphalt Concrete	*T*_0_ (°C)	*C*_1_	*C*_2_
20	1	1 × 10^−7^	5218.14	2240.85	20 (FAM)	25.4 (FAM)	233.2 (FAM)
	2	1 × 10^−6^	5381.72	3212.28	20 (AC)	25.7 (AC)	218.9 (AC)
	3	1 × 10^−5^	5499.81	3913.62	-	-	-
	4	1 × 10^−4^	5570.35	4897.11	-	-	-
	5	1 × 10^−3^	5112.13	5645.90	-	-	-
	6	1 × 10^−2^	3608.49	5520.75	-	-	-
	7	1 × 10^−1^	1489.17	3509.47	-	-	-
	8	1 × 10^0^	512.63	1405.91	-	-	-
	9	1 × 10^1^	154.93	531.44	-	-	-
	10	1 × 10^2^	55.72	171.11	-	-	-
	11	1 × 10^3^	13.97	68.48	-	-	-
	12	1 × 10^4^	6.79	18.48	-	-	-
Equilibrium modulus, *E*_e_	-	-	34.20	197.25	-	-	-

**Table 5:** Parameters of the models.

Material	Young’s Modulus (MPa)	Poisson’s Ratio	Coefficient of Thermal Expansion (10^−5^/°C)	Thermal Conductivity [(J/m·h·°C)]	Specific Heat [(J/kg·°C)]	Density (kg/m^3^)
Aggregate	80,000	0.20	1.20	5472	920	2600
FAM	805	0.25	10.52	4680	923	2200
AC-13	3592	0.35	2.19	5112	916	2340
Base layer	200	0.35	2.00	5616	912	2200
Subgrade	70	0.40	2.00	5616	1040	1800

## Data Availability

The original contributions presented in this study are included in the article. Further inquiries can be directed to the corresponding author.
